# Hospital-onset sepsis and community-onset sepsis in critical care units in Japan: a retrospective cohort study based on a Japanese administrative claims database

**DOI:** 10.1186/s13054-022-04013-0

**Published:** 2022-05-13

**Authors:** Mayuko Tonai, Atsushi Shiraishi, Toshiyuki Karumai, Akira Endo, Hirotada Kobayashi, Kiyohide Fushimi, Yoshiro Hayashi

**Affiliations:** 1 Department of Intensive Care Medicine, Kameda Medical Center, Kamogawa, Japan; 2 Emergency and Trauma Center, Kameda Medical Center, Kamogawa, Chiba 296-8602 Japan; 3 Trauma and Acute Critical Care Center, Tokyo Medical and Dental University Hospital, Tokyo, Japan; 4 Division of Renal Medicine, Brigham and Women’s Hospital, Boston, USA; 5 Department of Health Policy and Informatics, Tokyo Medical and Dental University Graduate School of Medicine, Tokyo, Japan; 6 Department of Acute Critical Care Medicine, Tsuchiura Kyodo General Hospital, Tsuchiura, Japan

**Keywords:** Intensive care unit, Organ support therapy, Mortality, Hospital length of stay, Resource utilization

## Abstract

**Background:**

Hospital- and community-onset sepsis are significant sepsis subgroups. Japanese data comparing these subgroups are limited. This study aimed to describe the epidemiology of hospital- and community-onset sepsis in critical care units in Japan.

**Methods:**

We performed a retrospective cohort study using the Japanese Diagnosis and Procedure Combination database. Adult patients admitted to critical care units with sepsis from April 2010 to March 2020 were included. Sepsis cases were identified based on ICD-10 codes for infectious diseases, procedure codes for blood culture tests, and medication codes for antimicrobials. Patients’ characteristics, in-hospital mortality, and resource utilization were assessed. The in-hospital mortality between groups was compared using the Poisson regression generalized linear mixed-effect model.

**Results:**

Of 516,124 patients, 52,183 (10.1%) had hospital-onset sepsis and 463,940 (89.9%) had community-onset sepsis. Hospital-onset sepsis was characterized by younger age, infrequent emergency hospitalization, frequent surgery under general anesthesia, and frequent organ support upon critical care unit admission compared to community-onset sepsis. In-hospital mortality was higher for hospital-onset than for community-onset sepsis (35.5% versus 19.2%; unadjusted mean difference, 16.3% [95% confidence interval (CI) 15.9–16.7]; adjusted mean difference, 15.6% [95% CI 14.9–16.2]). Mean hospital length of stay was longer for hospital-onset than for community-onset sepsis (47 days versus 30 days; unadjusted mean difference, 17 days [95% CI 16–17]; adjusted mean difference, 13 days [95% CI 12–14]).

**Conclusion:**

Patients with hospital-onset sepsis admitted to critical care units in Japan had a poorer prognosis and more resource utilization including organ support rate, number of days with critical care unit surcharge codes, and hospital length of stay than those with community-onset sepsis.

**Supplementary Information:**

The online version contains supplementary material available at 10.1186/s13054-022-04013-0.

## Background

Sepsis is a heterogeneous syndrome with varying characteristics and therapeutic outcomes depending on the geographical region, socioeconomic status, patients’ backgrounds, causative pathogens, anatomical sites of infection, host responses, and quality of care [1–3]. The heterogeneity of sepsis hinders clinical research planning, potentially limiting the discovery of effective treatment options for specific subgroups and making healthcare policymaking difficult [1, 4, 5].

A better understanding of the epidemiology of sepsis from the subgrouping by the location of onset, namely hospital-onset sepsis versus community-onset sepsis perspective, helps in planning future interventional studies of sepsis and devising healthcare policies [1, 3, 6, 7]. Some studies have demonstrated differences in epidemiology between hospital- and community-onset sepsis [8–11]. Patients with hospital-onset sepsis had more extended stays in the hospital and intensive care unit (ICU) than patients with community-onset sepsis; hospital-onset sepsis was associated with a twofold to threefold increased risk of mortality than community-onset sepsis in the USA [8, 9]. Costs of staying in the ICU were higher for hospital-onset sepsis than for community-onset sepsis in the USA and France [10, 11].

These characteristics might vary significantly from country to country, as each country has different healthcare systems, healthcare resources, and population compositions. Given that Japan has had the largest aging population worldwide since 2005, its original analysis may serve as crucial data for future clinical research and policymaking [12]. However, the nationwide epidemiology of sepsis admitted to critical care units primarily focusing on the difference between community- and hospital-onset sepsis has never been studied in Japan [13–16].

This study aimed to describe differences in characteristics and clinical outcomes between hospital- and community-onset sepsis admitted to critical care units in Japan using a national administrative claims database.

## Methods

### Study design and data source

The retrospective cohort study was conducted using the Japanese Diagnosis Procedure Combination (DPC) database. The DPC database is a nationwide discharge and administrative claims database in Japan. This database includes information on each hospitalization, consisting of the International Classification of Diseases, Tenth Revision (ICD-10) diagnostic codes, daily medical procedures, daily records of drugs administered and devices used, and the case fatality at hospital discharge. As for the diagnostic codes, up to six diagnosis codes at admission and four diagnostic codes after admission are coded by ICD-10. As of 2019, 1724 hospitals had contributed to the DPC database, which accounted for more than 80% of acute care hospital beds in Japan. The present study included data accumulated from April 2010 to March 2020. The study protocol was approved by the Institutional Review Board of Tokyo Medical and Dental University (approval number: M2000-788-26). The need for informed consent was waived on account of the anonymous nature of the data.

### Participants

We included adult patients who were admitted to critical care units with sepsis and met the following conditions: (1) age ≥ 18 years; (2) presence of ICD-10 codes for infectious diseases that could cause sepsis (Additional file 1: Appendix 1) at any time during hospitalization; (3) admission to critical care units including the ICU, high dependency unit (HDU, also known as the step-down unit or progressive care unit), and emergency ICU (EICU) (Additional file 1: Appendix 2); and (4) a combination of procedure codes for blood culture tests (Additional file 1: Appendix 3) and medication codes for antimicrobials (Additional file 1: Appendix 4) during 3 consecutive days before and after admission to the critical care unit (for patients admitted to the critical care unit on the first day of hospitalization, two consecutive days including the first and second days of admission). There were no exclusion criteria. The list of ICD-10 codes for infectious diseases that could cause sepsis (Additional file 1: Appendix 1) was generated using the Delphi method based on agreements among three independent intensivists (M.T., T.K., and H.K.).

### Variables

According to previous studies, variable data for the present study were extracted from the DPC database [17–23]. The ICD-10 codes and specific codes for reimbursement used in this study are described separately (Additional file 1: Appendix 1–7).

We collected data on age, sex, height, weight, emergency hospitalization, and Elixhauser comorbidity scores [24, 25] as baseline characteristics of patients at the time of hospitalization. We also collected data on the volumes of fluids, including crystalloids, colloids, and red blood cell products administered on the first and second days of admission to the critical care unit, organ support (use of vasopressors, mechanical ventilation, and renal replacement therapy) on the day of admission to the critical care unit, and diagnostic codes during hospitalization as clinical characteristics. Data on surgical procedures performed under general anesthesia during hospitalization were handled as intermediate variables. Data on the focus of infection were additionally collected as reference information. The study exposure variables were classified as hospital- and community-onset sepsis, defined as critical care unit admission after the third day of hospitalization and on the first or second day of hospitalization, respectively. This definition may lead to misclassification bias, and we conducted sensitivity analyses described later to reduce these biases. Severity scores such as APACHE-II and SOFA were not collected because the DPC database does not have this information.

The primary outcome was in-hospital mortality. The secondary outcomes included critical care unit mortality up to day 14, number of days applied with critical care unit charge up to 14 days, hospital length of stay, and the number of days required for organ support after critical care unit admission. The DPC database system had upper limits for obtaining critical care unit charges (ICU and EICU codes for up to 14 days and HDU codes for up to 21 days). Thus, the number of days applied with critical care unit charge was counted to 14 days adopting the shorter limit of the former. The codes for organ support are described separately (Additional file 1: Appendix 3, 5, 6).

### Statistical analysis

Continuous variables are presented as medians and inter-quartile ranges. Categorical variables are presented as numbers and percentages. The associations between hospital- or community-onset sepsis and outcomes were assessed using a Poisson regression generalized linear mixed-effect model adjusted for patient age, sex, and comorbidity as fixed-effect confounders and clustered by hospitals as random-effect confounders. Unadjusted and adjusted differences of outcomes were reported with 95% confidence intervals (CIs).

Subgroup analyses were performed to explore the impact of hospital- and community-onset sepsis on the primary outcomes of patients with the following seven characteristics: (1) admission to tertiary medical care centers; (2) admission to hospitals that have a critical care training unit accredited by The Japanese Society of Intensive Care Medicine (JSICM); (3) receipt of surgical procedures under general anesthesia during hospitalization; (4) use of vasopressors on the day of critical care unit admission; (5) use of mechanical ventilation on the day and the second day of critical care unit admission; (6) renal replacement therapy within 14 days after critical care unit admission; and (7) admission to critical care unit limited to ICUs.

In this study, inclusion criteria might lead to selection bias for the following reasons: First, a clinical definition of sepsis (i.e., Sepsis-3) could not be used because the DPC database does not contain variables to define this. Second, the DPC database contains a limited number of diagnostic codes by ICD-10. Third, the validity of the ICD-10 codes that may cause sepsis has not been verified. Therefore, we conducted a sensitivity analysis in the subset of patients with ICD-10 codes that directly indicated “sepsis” (Additional file 1: Appendix 7, sensitivity analysis #1). Since hospital- and community-onset sepsis were not defined based on a clinical definition, which leads to misclassification bias, we conducted sensitivity analyses using three definitions of the hospital- and community-onset sepsis. We defined hospital-onset as critical care unit admission after the fourth day of hospitalization and community-onset as critical care unit admission on the first or second day of hospitalization (sensitivity analysis #2). Furthermore, we defined hospital- and community-onset based on whether ICD-10 diagnoses of infectious diseases were encoded after or at the time of hospitalization (sensitivity analysis #3). Lastly, we defined hospital- and community-onset by the date of critical care unit admission and ICD-10 codes at hospitalization (sensitivity analysis #4). Specifically, hospital-onset was defined as “critical care unit admission after the third day of hospitalization” and “the ICD-10 diagnosis for an infectious disease was not encoded at the time of hospitalization,” and community-onset was defined as “critical care unit admission on the first or second day of hospitalization” and “the ICD-10 diagnosis for an infectious disease was encoded at the time of hospitalization.” Statistical analyses were performed using R, version 3.6.0 (R Development Core Team, Vienna, Austria). The Poisson regression generalized linear mixed-effect model was created and optimized using the lmerTest package for R [26]. All statistical analyses were performed on complete case analysis without missing data imputation. Analysis items with p < 0.05 were considered statistically significant.

## Results

**Fig. 1:**
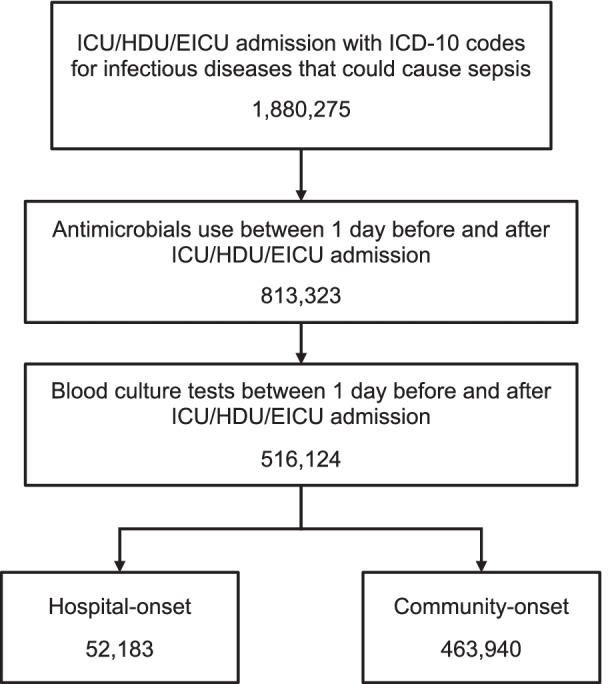
Flowchart of the study patients. *ICU* Intensive care unit, *HDU* High dependency unit, *EICU* Emergency intensive care unit

Of 1,880,275 patients admitted to the critical care units with ICD-10 codes for infectious diseases that could cause sepsis, 516,124 had relevant blood culture test codes and antimicrobial codes during the three calendar days between 1 day before and after critical care unit admission. These patients were considered as having sepsis (Fig. 1). Of those patients, 52,183 (10.1%) had hospital-onset sepsis and 463,940 (89.9%) had community-onset sepsis. A total of 1221 hospitals have requested critical care unit charge in this study.

Patients with hospital-onset sepsis were characterized by younger age, less frequent emergency hospitalization, more frequent concomitant surgical procedures under general anesthesia during hospitalization, and more frequent use of organ support on the day of critical care unit admission (Table 1). The hospital-onset sepsis group had a median of 10 days from the date of hospitalization to critical care unit admission. Respiratory was the most common source of infection in both groups (Table 1).

**Table 1:** Characteristics of patients

	Hospital-onset sepsis (*n* = 52,183)	Community-onset sepsis (*n* = 463,940)
Female sex	18,812 (36.1)	190,917 (41.2)
Age (year)	72 [63–79]	77 [66–84]
Weight (kg)	54.0 [45.6–63.2]	51.7 [42.0–61.6]
Body mass index (kg/m^2^)	21.6 [18.9–24.5]	21.5 [18.6–24.6]
Emergency hospitalization	29,134 (55.9)	416,535 (89.9)
Elixhauser index	5 [2–11]	4 [0–7]
Surgery under general anesthesia during hospitalization	25,568 (49.0)	85,070 (18.3)
Days from hospitalization to critical care unit admission, days	10 [5–22]	1 [1–1]
*Types of critical care unit*		
Intensive care unit	41,460 (79.5)	120,621 (26.0)
High dependency unit	10,485 (20.1)	83,257 (17.9)
Emergency intensive care unit	238 (0.5)	260,062 (56.1)
*Organ support on the day of critical care unit admission*		
Vasopressor	23,123 (44.3)	110,376 (23.8)
Mechanical ventilation	18,906 (36.2)	95,165 (20.5)
Renal replacement therapy	7510 (14.4)	25,479 (5.5)
Infusion fluid volume on the 1–2 days of critical care unit, ml	7620 [3600–15,430]	3800 [1660–8400]
Focus of infection*		
Respiratory	17,251 (33.1)	212,922 (45.9)
Abdominal	16,837 (32.3)	116,011 (25.0)
Urogenital	2004 (3.8)	50,600 (10.9)
CNS	1946 (3.7)	27,072 (5.8)
Cardiovascular	4771 (9.1)	26,607 (5.7)
Blood	3889 (7.5)	21,565 (4.6)
Bone and soft tissue	2205 (4.2)	21,641 (4.7)
Others	27,293 (52.3)	202,967 (43.7)

Data are presented as *n* (%) or median [IQR]. The medical codes of critical care units, vasopressor, mechanical ventilation, and renal replacement therapy are described separately (Additional file 1: Appendix 1–7)

*In cases where multiple ICD-10 codes were registered, all of them were counted as the focus of infection

**Fig. 2:**
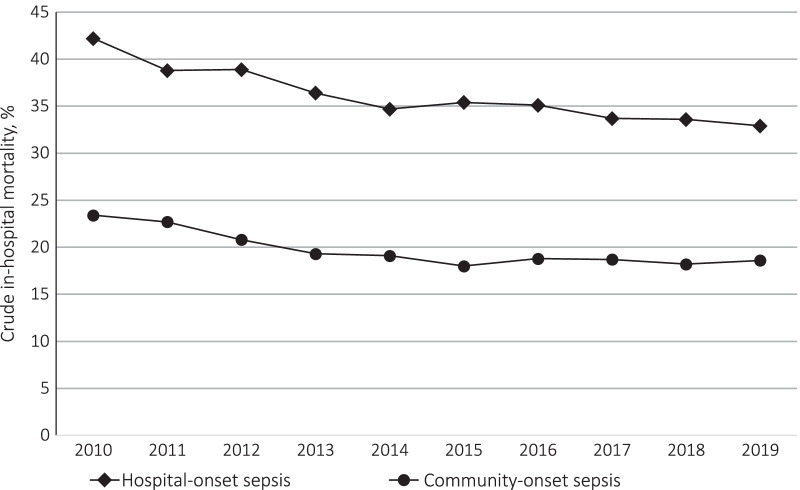
Trends in in-hopital mortality for the study patients from 2010 to 2019. The changes in in-hospital mortality of hospital- and community-onset sepsis from 2010 to 2019 are shown. The diamond-shaped mark indicates hospital-onset sepsis, and the circle mark indicates community-onset sepsis

The in-hospital mortality was 35.5% for the hospital-onset sepsis group and 19.2% for the community-onset sepsis group (unadjusted difference 16.3% [95% CI 15.9–16.7]) (Table 2). The difference was 15.6% after adjustment for age, sex, comorbidity, and clustering by the hospital (95% CI 14.9–16.2). In-hospital mortality has decreased over the 10 years in both groups (Fig. 2). The mean length of stay in hospital after critical care unit admission, the mean number of days applied with critical care unit charge up to 14 days, and the mean duration of organ support after critical care unit admission were all longer in the hospital-onset sepsis group than in the community-onset sepsis group (Table 2).

**Table 2:** Outcomes of hospital- and community-onset sepsis

	Hospital-onset sepsis (*n* = 52,183)	Community-onset sepsis (*n* = 463,940)	Unadjusted difference [95% CI]	Adjusted difference [95% CI]
*Primary outcome*				
In-hospital mortality	18,520 (35.5)	89,017 (19.2)	16.3 [15.9–16.7]	15.6 [14.9–16.2]
*Secondary outcomes*				
Critical care unit mortality	6675 (12.8)	35,035 (7.6)	5.2 [5.0–5.5]	5.6 [5.2–6.0]
Hospital LOS	47 (61)	30 (36)	17 [16, 17]	13 [12–14]
Number of days applied with critical care charge	6 (5)	5 (4)	1 [1–1]	1 [1–1]
Days on vasopressor	4 (9)	2 (5)	2 [2–2]	1 [1–1]
Days on MV	9 (26)	4 (14)	5 [5–5]	4 [3, 4]
Days on RRT	3 (11)	1 (6)	2 [2–2]	1 [1–1]

Data are presented as n (%) or mean (S.D.). 95% CI indicates confidence interval

*LOS* Length of stay, *MV* Mechanical ventilation, *RRT* Renal replacement therapy

**Fig. 3:**
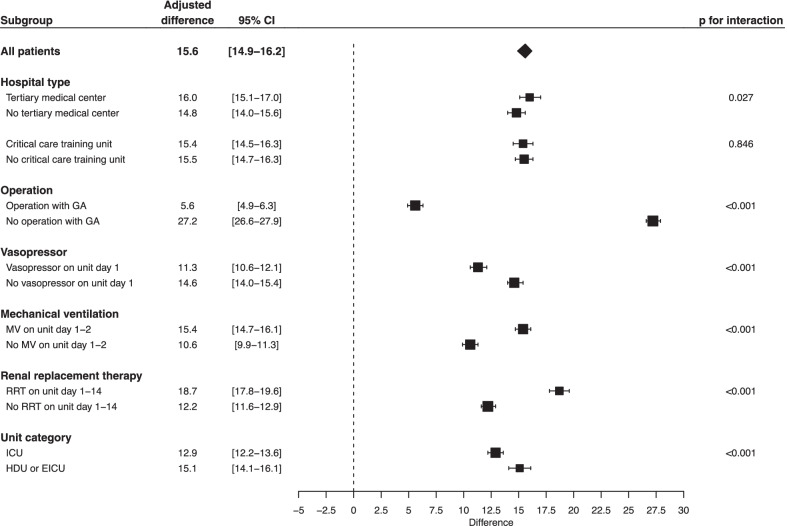
Subgroup analyses. Subgroup analyses with adjusted mean differences and 95% confidence intervals for in-hospital mortality adjusted by age, sex, and Elixhauser comorbidity score, and stratified by hospital. *CI* Confidence interval, *GA* General anesthesia, *MV* Mechanical ventilation, *RRT* Renal replacement therapy, *ICU* Intensive care unit, *HDU* High dependency unit, *EICU* Emergency intensive care unit

In the subgroup analyses, there were statistically significant interactions between in-hospital mortality and the receipt of surgical procedures during hospitalization, use of vasopressors on the first day of critical care unit admission, use of ventilator at the early stage of critical care unit stay, and receipt of renal replacement therapy from the day of critical care unit admission to day 14 (Fig. 3). Notably, in the subgroup of patients who did not undergo surgical procedures under general anesthesia during hospitalization, there was a notable difference in in-hospital mortality between those with hospital-onset sepsis and those with community-onset sepsis; the in-hospital mortality was 49.4% among patients with hospital-onset sepsis and 20.4% among those with community-onset sepsis, and the adjusted difference was 27.2% (95% CI 26.6–27.9) (Fig. 3). Admission to a tertiary medical care center and admission to a hospital that has JSICM-accredited critical care training unit did not show significant interaction with in-hospital mortality. In all the sensitivity analyses, the in-hospital mortality was higher among patients with hospital-onset sepsis than among those with community-onset sepsis, as observed in the primary analysis (Table 3).

**Table 3:** Sensitivity analyses of in-hospital mortality

	Hospital-onset sepsis	Community-onset sepsis	Unadjusted difference [95% CI]	Adjusted difference [95% CI]
Analysis #1^a^	*n* = 3912	*n* = 122,364		
In-hospital mortality	1610 (41.2)	30,219 (24.7)	16.5 [15.1–17.8]	15.5 [13.8–17.1]
Analysis #2^b^	*n* = 45,054	*n* = 463,940		
In-hospital mortality	16,783 (37.3)	89,017 (19.2)	18.1 [17.7–18.5]	17.2 [16.6–17.9]
Analysis #3^c^	*n* = 118,039	*n* = 398,085		
In-hospital mortality	28,826 (24.4)	78,712 (19.8)	4.6 [4.4–4.9]	3.6 [3.2–4.0]
Analysis #4^d^	*n* = 26,266	*n* = 372,168		
In-hospital mortality	9470 (36.1)	69,662 (18.7)	17.3 [16.8–17.8]	16.0 [15.1–16.8]

Data are presented as n (%). 95% CI indicates confidence interval

^a^Analysis of the subset of patients with ICD-10 codes that directly indicated “sepsis”

^b^Analysis with the definition of hospital-onset as critical care unit admission after the fourth day of hospitalization and community-onset as critical care unit admission on the first or second day of hospitalization

^c^Analysis with the definition of hospital- and community-onset according to whether the ICD-10 diagnoses of infectious diseases were coded at the time of hospitalization

^d^Analysis with the definition of hospital- and community-onset by the date of critical care unit admission and ICD-10 codes at the time of hospitalization

## Discussion

In the present study of data from a Japanese national administrative claims database, we found that patients with hospital-onset sepsis were younger, had higher comorbidity scores, and were likelier to have undergone surgical procedures during hospitalization than those with community-onset sepsis. Besides, patients with hospital-onset sepsis had higher mortality and greater resource utilization rates, including organ support, critical care unit stay, and hospital stay, than those with community-onset sepsis. The present study results were robust because of their high internal validity based on multiple sensitivity analyses.

Previous studies showed that hospital-onset sepsis resulted in higher mortality than community-onset sepsis [8–11, 27–31]. Similarly, the present study demonstrated that the in-hospital mortality of hospital-onset sepsis was nearly double that of community-onset sepsis (35.5% vs 19.2%). A higher comorbidity index score could explain the poor prognosis of hospital-onset sepsis, depending on conditions associated with therapeutic intervention before critical care unit admission and changes in the immune capacity [9, 32, 33]. Moreover, other unadjusted factors, such as the prevalence of multi-drug resistant pathogens and the time to resuscitation, including administration of antimicrobial agents, which is said to be longer in the general ward than in the emergency department for underrecognition of sepsis or resource shortage, may also have influenced the incidence of worse outcomes in hospital-onset sepsis than in community-onset sepsis [34–37]. In the subgroups analyses, differences in in-hospital mortality between hospital- and community-onset sepsis were widened among subgroups of patients who received surgical procedures under general anesthesia during hospitalization, those who applied mechanical ventilation in the early stages of critical care unit stay, and those who received RRT within 14 days of admission to the critical care unit. It can be hypothesized that the higher in-hospital mortality than community-onset sepsis can be explained by the higher severity of the disease.

The present study had some strengths. First, it is the world’s most extensive epidemiological study of the hospital- and community-onset sepsis to date. Moreover, it is the most comprehensive nationwide study conducted in Japan focused on hospital- and community-onset sepsis admitted to critical care units. As of 2019, the DPC database covered more than 80% of acute care hospital beds in Japan. Second, this study showed the impact of hospital-onset sepsis in critical care units of the most aging society [38]. In the present study, the median age of patients was more than 70 years, which was higher than those reported in previous studies in which the mean or median ages of the study population were in the 60s [8, 9, 11, 27]. Third, the present study showed that the duration of renal replacement therapy, mechanical ventilation, and vasopressor use in hospital- and community-onset sepsis were all longer in hospital-onset sepsis than that in community-onset sepsis. Previously, the Extended Prevalence of Infection in Intensive Care (EPIC) I, II and III studies and the Sepsis Occurrence in Acutely Ill Patients (SOAP) study showed the international epidemiology and clinical outcomes of hospital-onset sepsis [20, 23, 39, 40]. However, no study has explicitly presented the duration of organ support in hospital-onset sepsis compared to that in community-onset sepsis. The results of the present study provide new insights into the resource usage for hospital-onset sepsis. The occurrence of sepsis during hospitalization requires additional medical resources, including ICU and hospital beds.

Conversely, the present study had several limitations. First, our definition of sepsis was different from the Sepsis-3 definition because the DPC database did not contain enough information required to diagnose sepsis based on the Sepsis-3 criteria. Moreover, the validity of the definitions used in this study has not been verified. Second, although several sensitivity analyses were performed, misclassifications could have occurred between hospital-onset sepsis and community-onset sepsis. Third, some important subgroups of sepsis, such as ICU-onset sepsis and healthcare-onset sepsis, such as in-home or nursing home healthcare-related sepsis, could not be identified in this study. Fourth, variables related to infection represented by the results of culture tests, the prevalence of multi-drug resistant pathogens, and the appropriateness of antibiotic selection were not evaluated. Fifth, the DPC database does not include severity scores, and it was technically difficult to adjust the severity using alternative data. Severity is essential at the time of the occurrence of the hospital- or community-onset exposure, so it may not be appropriate to adjust this. Sixth, the critical care unit mortality can be underestimated because the DPC database system has upper limits on the number of days applied with critical care unit charge. Also, although the number of days applied with critical care charge was obtained, exact data on critical care unit length of stay could not be obtained. Seventh, the present study results cannot be applied directly to patients in other countries, as this study only evaluated patients in a Japanese database. Finally, there are unknown confounding factors that were not adjusted.

## Conclusions

Patients with hospital-onset sepsis admitted to critical care units in Japan had a poorer prognosis and more resource utilization, including organ support, the number of days applied with critical care unit charge, and hospital stay than those with community-onset sepsis. The epidemiology of hospital- and community-onset sepsis in Japan, a country with the most aging populations, can serve as primary data for future clinical research and healthcare policymaking.

## Supplementary Information

**Additional file 1: Appendix 1**. List of ICD-10 codes for infectious disease with presumed focus. **Appendix 2**. Critical care unit codes. **Appendix 3**. Procedure codes. **Appendix 4**. Antimicrobial codes. **Appendix 5**. Vasopressor codes. **Appendix 6**. Fluid codes. **Appendix 7**. ICU-10 codes directly indicate “sepsis.”

## Data Availability

The datasets analyzed in the current study are not publicly available for potential concerns of leakage of personally identifiable information.

## Abbreviations

ICU: Intensive care unit
DPC: Diagnosis procedure combination
ICD-10: International Classification of Diseases, Tenth Revision
HDU: High dependency unit
EICU: Emergency intensive care unit
CI: Confidence interval
JSICM: Japanese Society of Intensive Care Medicine
