# Supplementary material for: Hospital-onset sepsis and community-onset sepsis in critical care units in Japan: a retrospective cohort study based on a Japanese administrative claims database
Source: Crit Care. 2022 May 13;26:136. doi: 10.1186/s13054-022-04013-0 (PMC9107171; doi:10.1186/s13054-022-04013-0)
Supplement: Supplementary file 1 — Additional file 1: Appendix 1. List of ICD-10 codes for infectious disease with presumed focus. Appendix 2. Critical care unit codes. Appendix 3. Procedure codes. Appendix 4. Antimicrobial codes. Appendix 5. Vasopressor codes. Appendix 6. Fluid codes. Appendix 7. ICU-10 codes directly indicate “sepsis.” [file 13054_2022_4013_MOESM1_ESM.docx]

Appendix

Appendix 1. List of ICD-10 codes for infectious disease with presumed focus

| **Focus** | **ICD-10 codes** |
| --- | --- |
| Abdominal | A00, A00.0, A00.1, A00.9, A01, A01.0-A01.4, A02, A02.0-02.2, A02.8, A02.9, A03, A03.0-A03.3, A03.8, A03.9, A04, A04.x, A05, A05.0-A05.4, A05.8, A05.9, A06, A06.x, A07, A07.0-A07.3, A07.8, A07.9, A08, A08.0-A08.5, A08.5a, A08.5b, A09, A18.3, A18.7, A21.3, A22.2, A42.1, A51.1, A54.6, A56.3, B05.4, B15, B15.0, B15.9, B16, B16.0-B16.2, B16.9, B17, B17.0-B17.2, B17.8, B18, B18.0-B18.2, B18.8, B18.9, B19, B19.0, B19.9, B25.1, B25.2, B26.3, B46.2, B51.0, B55.0, B57.3, B58.1, B65.1, B66.1, B66.3, B67.0, B67.5, B67.8, B70.0, B71.1, B76.0, B77.0, B78.0, B81, B81.1, B81.3, B81.4, B81.8, B82, B82.0, B82.9, D73.3, K35, K35.0, K35.1, K35.9, K36, K37, K51.5, K57.0, K57.2, K57.4, K57.8, K60.3-K60.5, K61, K61.0-K61.4, K63.0, K63.1, K65, K65.0, K65.8, K65.9, K67, K67.0-K67.3, K67.8, K75.0, K77.0, K80.0, K80.1, K80.3, K80.4, K81, K81.0, K81.1, K81.8, K81.9, K82.2, K82.3, K83.0, K83.2, K83.3, O98.4, P35.3, P78.0, P78.1 |
| Blood | A19, A19.0-A192, A19.8, A19.9, A39.2-A39.4, A40.0, A49.0, A49.1, A68, A68.0, A68.1, A68.9, A79.0 |
| Bone and soft tissue | A18.0, A18.2, A18.4, A20.1, A22.0, A26, A26.0, A26.7-A26.9, A28.1, A30, A30.0-A30.5, A30.8, A30.9, A31.1, A32.0, A36.3, A42.2, A43.1, A44.1, A46, A48.0, A54.4, A60.1, A66, A66.0, A66.7, A66.9, A69.0, A69.1, B00.1, B01, B01.8, B01.9, B02, B08, B08.0, B08.2-B08.4, B08.8, B09, B42.1, B43.2, B45.3, B67.2, B70.1, B78.1, H60, H60.0-H60.3, H60.8, H60.9, H61.0, H62.0-H62.3, H70.0-H70.2, H70.8, H70.9, I89.1, K04.0, K04.6, K04.7, K10.3, K12.2, L00, L01, L01.0, L01.1, L02, L02.0-L02.4, L02.8, L02.9, L03, L03.0-L03.3, L03.8, L03.9, L04, L04.0-L04.3, L04.8, L04.9, L05.0, L08, L08.0, L08.8, L08.9, L30.3, L66.3, L73.2, L88, L98.0, M00, M00.0-M002, M00.8, M00.9, M01, M01.0-M01.6, M01.8, M46.2, M46.3, M46.5, M49.0-M49.3, M60.0, M63.0-M63.2, M65.0, M65.1, M68.0, M71.0, M71.1, M72.6, M73.0, M73.1, M86, M86.0-M86.6, M86.8, M86.9, M90.0-M90.2, O86.0, O91, O91.0, O91.1, P39.0, P39.4, T79.3, T84.5, T84.7, T87.4 |
| Cardiovascular | A39.5, A52.0, B33.2, B37.6, B57.2, I30, I30.1, I30.8, I30.9, I32.0, I32.1, I33, I33.0, I33.9, I38, I40, I40.0, I40.1, I40.8, I40.9, I41.0-I41.2, I43.0, I52.0, I52.1, I80, I80.0-I80.3, I80.8, I80.9, I82.1, I98.0, I98.1, K75.1, O22.2, O87.0, T82.6, T82.7 |
| Central nervous system | A17, A17.0, A17.1, A17.8, A17.9, A20.3, A32.1, A33, A34, A35, A39.0, A52.1, A52.3, A80, A80.0-A80.4, A80.9, A81, A81.0-A81.2, A81.8, A81.9, A82, A82.0, A82.1, A82.9, A83, A83.0-A83.6, A83.8, A83.9, A84, A84.0, A84.1, A84.8, A84.9, A85, A85.0-A85.2, A85.8, A86, A87, A87.0-A87.2, A87.8, A87.9, A88, A88.0, A88.8, A89, B00.3, B00.4, B01.0, B01.1, B02.0-B02.2, B05.0, B05.1, B06.0, B26.1, B26.2, B37.5, B38.4, B43.1, B45.1, B46.1, B50.0, B57.4, B58.2, B69.0, B83.2, G00, G00.0-G00.3, G00.8, G00.9, G01, G02, G02.0, G02.1, G02.8, G03, G03.1, G03.2, G03.8, G03.9, G04, G04.0, G04.2, G04.8, G04.9, G05, G05.0-G05.2, G05.8, G06, G06.0-G06.2, G07, G08, I68.1 |
| Respiratory | A15, A15.x, A16, A16.0-A16.5, A16.7-A16.9, A20.2, A21.2, A22.1, A31.0, A36.0-A36.2, A37, A37.0, A37.1, A37.8, A37.9, A42.0, A43.0, A48.1, A48.2, A54.5, A56.4, A70, B00.2, B01.2, B05.2, B08.5, B20.6, B25.0, B30.2, B33.4, B37.1, B38.0-B38.2, B39.0-B39.2, B40.0-B40.2, B41.0, B42.0, B44.0, B44.1, B45.0, B46.0, B58.3, B66.4, B67.1, B83.3, B96.0, J00, J01, J01.0-J01.4, J01.8, J01.9, J02, J02.0, J02.8, J02.9, J03, J03.0, J03.8, J03.9, J04, J04.0-J04.2, J05, J05.0, J05.1, J06, J06.0, J06.8, J06.9, J10, J10.0, J10.1, J10.8, J11, J11.0, J11.1, J11.8, J12, J12.0-J12.2, J12.8, J12.9, J13, J14, J15, J15.x, J16, J16.0, J16.8, J17, J17.0-J17.3, J17.8, J18, J18.0-J18.2, J18.8, J18.9, J20, J20.x, J21, J21.0, J21.8, J21.9, J22, J31, J31.0-J31.2, J32, J32.0-J32.4, J32.8, J32.9, J34.0, J35.0, J36, J37, J37.0, J37.1, J39.0, J39.1, J40, J41, J41.0, J41.1, J41.8, J42, J85, J85.1-J85.3, J86, J86.0, J86.9, O74.0, P23, P23.0-P23.6, P23.8, P23.9, R09.1, U04, U04.9 |
| Urogenital | A18.1, A51, A51.0, A54.0-A54.2, A56.0-A56.2, A59.0, A60.0, B26.0, B37.4, B52.0, N11.0, N11.1, N13.6, N15.1, N30, N30.0, N33.0, N34.0, N39.0, N41.0-N41.3, N43.1, N45, N45.0, N45.9, N70, N70.0, N70.1, N70.9, N71, N71.0, N71.1, N71.9, N72, N73, N73.0-N73.6, N73.8, N73.9, N74, N74.0-N74.4, N74.8, N75.1, N76.0-N76.4, N77.0, N77.1, O03.0, O03.5, O04.0, O04.5, O05.0, O05.5, O06.0, O06.5, O07.0, O07.5, O08.0, O23, O23.0-O23.5, O23.9, O86.1-O86.3, P39.3, T83.5, T83.6 |
| Others / Unknown | A18, A18.5, A18.6, A18.8, A20, A20.0, A20.7-A20.9, A21, A21.0, A21.1, A21.7-A21.9, A22, A22.7-A22.9, A23, A23.0-A23.3, A23.8, A23.9, A24, A24.0-A24.4, A25, A25.0, A25.1, A25.9, A27, A27.0, A27.8, A27.9, A28, A28.0, A28.2, A28.8, A28.9, A31, A31.8, A31.9, A32, A32.7-A32.9, A36, A36.8, A36.9, A38, A39, A39.1, A39.8, A39.9, A40, A40.1-A40.3, A40.8, A40.9, A41, A41.0-A41.5, A41.8, A41.9, A42, A42.7-A42.9, A43, A43.8, A43.9, A44, A44.0, A44.8, A44.9, A48, A48.3, A48.4, A48.8, A49, A49.2, A49.3, A49.8, A49.9, A50, A50.0-A50.2, A50.9, A51.2, A51.4, A51.5, A51.9, A52, A52.7, A53, A53.0, A53.9, A54, A54.8, A54.9, A55, A56, A56.8, A59, A59.8, A59.9, A60, A60.9, A63, A63.8, A64, A65, A69, A69.2, A69.8, A69.9, A74, A74.8, A74.9, A75, A75.0-A75.3, A75.9, A77, A77.0-A77.3, A77.8, A77.8a, A77.8b, A77.9, A78, A79, A79.1, A79.8, A79.9, A90, A91, A92, A92.0-A92.4, A92.8, A92.9, A93, A93.0-A93.2, A93.8, A94, A95, A95.0, A95.1, A95.9, A96, A96.0-A96.2, A96.8, A96.9, A98, A98.0-A98.5, A98.8, A99, B00, B00.5, B00.7-B00.9, B02.3, B02.7-B02.9, B03, B04, B05, B05.3, B05.8, B05.9, B06, B06.8, B06.9, B20, B20.0-B20.5, B20.7-B20.9, B22, B23, B23.0, B23.8, B24, B25, B25.8, B25.9, B26, B26.8, B26.9, B27, B27.0, B27.1, B27.8, B27.9, B30, B30.0, B30.1, B30.3, B30.8, B30.9, B33, B33.0, B33.1, B33.3, B33.8, B34, B34.0-B34.4, B34.8, B34.9, B37, B37.7-B37.9, B38, B38.7-B38.9, B39, B39.3-B39.5, B39.9, B40, B40.7-B40.9, B41, B41.7-B41.9, B42, B42.7-B42.9, B43, B43.8, B43.9, B44, B44.2, B44.7-B44.9, B45, B45.7-B45.9, B46, B46.4, B46.5, B46.8, B46.9, B48, B48.2-B48.4, B48.7, B48.8, B49, B50, B50.8, B50.9, B51, B51.8, B51.9, B52, B52.8, B52.9, B53, B53.0, B53.1, B53.8, B54, B55, B55.9, B56, B56.0, B56.1, B56.9, B57, B57.0, B57.1, B57.5, B58, B58.8, B58.9, B59, B60, B60.0-B60.2, B60.8, B64, B65, B65.0, B65.2, B65.8, B65.9, B66, B66.0, B66.2, B66.5, B66.8, B66.9, B67, B67.3, B67.4, B67.6, B67.7, B67.9, B68, B68.0, B68.1, B68.9, B69, B69.8, B69.9, B70, B71, B71.0, B71.8, B71.9, B72, B73, B74, B74.0-B74.4, B74.8, B74.9, B75, B76, B76.1, B76.8, B76.9, B77, B77.8, B77.9, B78, B78.7, B78.9, B79, B83, B83.0, B83.1, B83.8, B83.9, B89, B95, B95.0-B95.8, B96, B96.1-B96.8, B97, B97.0-B97.8, B99, E32.1, H05.0, H65.0, H65.2, H65.3, H66, H66.0-H66.4, H66.9, H67.0, H67.1, H68, H68.0, H73.0, H73.1, H75.0, H83.0, K11.2, K11.3, O75.3, O85, O86, O86.8, O88.3, O98, O98.0-O98.3, O98.5, O98.6, O98.8, O98.9, P35, P35.0-P35.2, P35.8, P35.9, P36, P36.0-P36.5, P36.8, P36.9, P37, P37.0-P37.5, P37.8, P37.9, P39, P39.2, P39.8, P39.9, T80.2, T81.4, T84.6, T85.7, T88.0 |

Appendix 2. Critical care unit charge codes

| **Types of critical care units** | **DPC codes** |
| --- | --- |
| ICU | 190174510, 190174410, 190174710, 190174610, 190174910, 190174810, 190116310, 190116410, 190139810, 190140010, 190140110, 190139910, 193005910, 193006410, 193010410, 193010710, 193010310, 193001610, 193010010, 193010110, 193010210, 193006210, 193001710, 193010610, 193010510, 193006110, 193006310, 193006010, 193507810, 193511810, 193511710, 193501610, 193501710, 193512010, 193508010, 193507910, 193512410, 193507610, 193507710, 193512310, 193512210, 193512110, 193507510, 193511910, 193307510, 193301510, 193310810, 193307410, 193307710, 193310410, 193310510, 193310710, 193310310, 193310610, 193307610, 193310210, 193310910, 193301410, 193307310, 193307210 |
| HDU | 190175010, 190175110, 193011110, 193010810, 193011010, 193010910, 193512510, 193512810, 193512610, 193512710, 193311010, 193311310, 193311210, 193311110, 190117310, 193003110, 193003010, 193501910, 193501810, 193301810, 193301910 |
| EICU | 190128610, 190024510, 190074510, 190128710, 190024310, 190024410, 190138510, 190138610, 190138310, 190138210, 190138410, 190138110, 190138710, 190138810, 190139310, 190139210, 190138910, 190139110, 190139010, 193004810, 193005110, 193000910, 193000610, 193004610, 193005010, 193003910, 193005410, 193003310, 193004010, 193004210, 193001210, 193004410, 193000310, 193004910, 193004110, 193004510, 193003410, 193005210, 193004710, 193004310, 193005310, 193505810, 193506610, 193500910, 193506010, 193511570, 193511670, 193506810, 193506410, 193506210, 193505510, 193504710, 193501210, 193506910, 193507010, 193505710, 193506510, 193506710, 193506110, 193505910, 193504610, 193500310, 193500610, 193505610, 193506310, 193306710, 193306610, 193306410, 193300110, 193306010, 193305710, 193305810, 193305410, 193304410, 193306210, 193306110, 193305210, 193300710, 193300410, 193305910, 193304310, 193306510, 193306310, 193305610, 193305510, 193305310, 193301010 |

Appendix 3. Procedure codes

| **Types of procedures** | **DPC codes** |
| --- | --- |
| Blood culture test | 160058610 |
| Ventilator support | 140009310, 140023510, 140010150, 140024350 |
| Renal replacement therapy | 140051110, 140051010, 140036710, 140052810, 140007710, 140029850 |

Appendix 4. Antimicrobial codes

| **Types of antimicrobials** | **DPC codes** |
| --- | --- |
| antibacterial agent | 620005060, 640463089, 620000427, 620006026, 620006027, 620006165, 620007537, 622329900, 621062901, 620006341, 620007538, 621380904, 621380908, 622330000, 620006342, 622329800, 621977300, 620006344, 620006343, 620006224, 620006261, 620006348, 620006348, 620008810, 621064601, 622111302, 621064701, 621064501, 620006225, 620006262, 620006349, 620006349, 620008811, 621065101, 621065201, 621065301, 621065001, 620006226, 620006227, 620003894, 620003894, 620003894, 620006791, 620008047, 620009408, 621738802, 640463091, 640463091, 620002907, 620002907, 620005694, 620005695, 620005695, 620006792, 620009575, 620009576, 621487601, 621487701, 621487802, 622107901, 622107901, 621942602, 621971901, 620004729, 620009511, 621876001, 621735001, 620004730, 620004730, 620009512, 621066501, 621066401, 620004728, 620009509, 621924501, 621896701, 620007432, 621955601, 621950502, 621936302, 620004709, 620004709, 620008212, 620008213, 620008532, 620008532, 620008531, 620008531, 620008533, 620008534, 620008534, 620008535, 620009572, 620009572, 620009571, 621995201, 622227801, 621901402, 621884501, 620009574, 620009574, 622216601, 622207201, 622227901, 622338101, 622085501, 640462005, 640462005, 620006477, 646130274, 646130275, 620008598, 620008599, 620008600, 640453139, 640453140, 646120062, 620003676, 621069302, 621069501, 622326000, 620003190, 621069402, 621069001, 620003462, 620003677, 621070102, 621070301, 620003191, 621070202, 621069701, 621070602, 621070902, 640453078, 620003198, 620003193, 620005641, 621604701, 646120080, 646120081, 640453079, 620005642, 621695601, 622213301, 620009196, 620008618, 620008753, 620008752, 622415701, 620003657, 620003658, 616120011, 616120012, 622199101, 610454003, 620006920, 620006920, 620007026, 620007026, 620007026, 622066601, 622112902, 620005906, 621073001, 616130132, 616130295, 616130039, 620007024, 622054901, 622066501, 622111101, 622127801, 622165902, 616130040, 620006829, 620006829, 620006829, 620006919, 620007025, 620008584, 620009117, 621073901, 621074701, 621075101, 621075101, 610451023, 621076301, 621076401, 621076401, 621076601, 621076801, 621077201, 621077501, 646130136, 620007456, 621078403, 621078301, 621078103, 621078601, 621342601, 646130137, 620007457, 621079203, 621079101, 621078903, 621079401, 621342802, 640462059, 622079701, 640462060, 622079801, 616130002, 621083104, 622316900, 616130378, 616130378, 616130003, 620003645, 621083503, 610454041, 610454042, 620004512, 620004981, 610453048, 610454043, 620003646, 616130115, 616130229, 620003647, 621085602, 621085602, 621087903, 622316800, 616130112, 621086806, 621087001, 621088403, 616130469, 616130110, 620008657, 621088802, 621089701, 621089501, 621088705, 621089103, 621089103, 621088901, 620005947, 616130404, 616130404, 616130404, 616130404, 610453059, 620000061, 616130405, 616130405, 616130405, 616130406, 616130406, 616130406, 620002756, 620002756, 620003583, 621675801, 616130407, 616130407, 616130408, 616130408, 620004958, 620004958, 620003968, 620003968, 620003968, 620003968, 620003970, 620008681, 620008681, 620008681, 621744203, 621744401, 621744401, 621747601, 620008682, 620008682, 621747701, 616130476, 616130476, 616130477, 620004513, 620004513, 621935801, 620008481, 620008483, 620008483, 620008483, 620008483, 620008482, 620008482, 620008482, 620008484, 620009343, 620009343, 620009344, 621889403, 621885302, 621935901, 620008487, 620008488, 620008489, 620008490, 616130531, 620008491, 620009334, 620009335, 620009335, 620009337, 620009337, 620009336, 621910002, 621885102, 616130532, 616130532, 620008492, 620009339, 620009340, 620009340, 620009342, 620009342, 620009342, 620009341, 621910102, 621885202, 620005503, 620005503, 620005501, 620005502, 620005499, 620005499, 620005500, 620008746, 620004080, 620005506, 620005506, 620005505, 620005507, 620005508, 610411059, 621955901, 621939401, 621940201, 621940201, 621940201, 621940201, 621940201, 621946301, 621946301, 621946301, 621931001, 621931001, 621962401, 621962501, 621942904, 610411057, 620009331, 621956001, 621956001, 621930801, 621940301, 621947501, 621947501, 621962601, 621943004, 610411058, 620009332, 621956101, 621956101, 621930901, 621940401, 621940401, 621940401, 621940401, 621947601, 621947601, 621947601, 621962701, 621943104, 646130121, 646130110, 646130122, 620004140, 620007293, 620007293, 620007293, 621989702, 621987602, 621924001, 646130111, 646130123, 620004141, 620007294, 620007294, 620007294, 620007294, 621989802, 621344901, 621924101, 646130112, 646130124, 620003732, 620007295, 620007295, 620007295, 620007295, 621345002, 621345301, 621095002, 621095002, 640408149, 620009563, 621540002, 640408148, 620003735, 622104201, 622130801, 622177601, 622124902, 622033402, 622104301, 622177701, 622328100, 620003736, 621096002, 620003737, 621096701, 621096503, 621096802, 621096601, 620003738, 621097303, 622177801, 621097602, 621097401, 620003733, 620003734, 620003740, 620003739, 620007318, 621757001, 621757001, 620005674, 620005675, 646130072, 646130073, 622125001, 622104001, 622131001, 622111501, 646130074, 621987501, 622104101, 622131101, 622111601, 646130075, 620006243, 620007540, 621101802, 621102102, 646130076, 620006244, 620007541, 621101902, 621102802, 640470010, 620004151, 621951701, 640454018, 620004652, 640454019, 620004653, 646130067, 646130067, 646130071, 646130132, 646130133, 646130134, 620002999, 620002999, 620002999, 620002999, 620002999, 620003827, 620006701, 620006701, 621967001, 621936401, 622052901, 622124801, 621756202, 621755802, 621755802, 621755802, 620003000, 620003000, 620003000, 620003000, 620003000, 620003001, 620003828, 620005676, 620006702, 620006702, 621967101, 621703001, 621756502, 621755902, 621755902, 621755902, 621755902, 646130268, 640463134, 640463134, 640463134, 640463134, 640463134, 620009566, 620009566, 620009567, 621987301, 621967202, 621538203, 622077302, 621836501, 646130269, 640463135, 640463135, 640463135, 640463135, 640463135, 640463080, 640463080, 640463080, 620009568, 621987401, 621488601, 621538303, 621555202, 621555101, 640470011, 620002955, 620002955, 620002955, 622051701, 620007514, 620007514, 620004662, 620004148, 620004148, 620004148, 620004148, 620004148, 620006706, 620006706, 620004149, 620004149, 620004149, 620004149, 620004149, 620006707, 620006707, 640407076, 621966801, 621966801, 621994601, 621994601, 640407077, 621966901, 621966901, 621994701, 621994701, 640407080, 640407081, 640443046, 640443047, 666130005, 666130005, 666130006, 666130006, 646130037, 646130301, 646130302, 640443048, 620005201, 620005202, 620005180, 620003780, 620003781, 646120011, 622037301, 622074801, 621111803, 622327500, 620003210, 620006210, 646120012, 620006211, 621112004, 616130512, 616130513, 621946401, 621964002, 620008731, 620008731, 621113002, 616130332, 621113712, 622323800, 616130526, 616130333, 622079901, 622329200, 620003815, 622104401, 622131501, 621812201, 622045702, 620003816, 620004767, 620004750, 620006316, 621766301, 621114602, 620003817, 620004768, 620004751, 620006317, 621766401, 621115302, 620007477, 640451022, 640451023, 610411055, 610411055, 620003004, 620003004, 620003004, 620003004, 620003004, 620004133, 620004133, 620004133, 620004133, 620004133, 620004135, 620005875, 620009561, 622250001, 622419601, 622408701, 620004775, 620004776, 620008446, 620008447, 622423101, 610411056, 620008702, 621926801, 621926801, 621116201, 621116301, 621709001, 620007519, 620009585, 620009586, 621952301, 621950101, 621967801, 621967901, 621946701, 621947901, 622044201, 620007520, 620009587, 620009588, 621952401, 621950201, 621968001, 621968101, 621946801, 621948001, 622044301, 622439001, 622419401, 620007518, 621952501, 621967701, 621931801, 640451036, 640451037, 621708501, 622078501, 621727601, 646130264, 620004152, 620007316, 620007316, 620007316, 620007316, 620008216, 620008216, 622083601, 622099301, 622130901, 622111401, 622080002, 646130265, 640444050, 640444051, 640444051, 640444071, 640444071, 640453097, 620003742, 620003742, 620003742, 620004714, 620004714, 620007317, 620007317, 620007317, 620007317, 621347901, 621116801, 621441901, 620004108, 620004108, 620007364, 620008211, 622010201, 622127601, 622172601, 620003703, 620003703, 620003703, 620004106, 620004155, 620004155, 620004707, 620005643, 620007365, 620007362, 620007363, 646130368, 646130369, 640406222, 620003003, 620003003, 620003003, 620003003, 620003003, 620004132, 620004132, 620004132, 620004132, 620004134, 620005874, 620009560, 622118402, 622118402, 622100601, 640406223, 620002977, 620002977, 620003002, 620000022, 620000022, 620001975, 620001975, 620001974, 620001974, 620004463, 620000020, 620000020, 620000021, 620000021, 621120001, 621120001, 620003495, 620003495, 620003496, 620003496, 620003554, 620003555, 620003555, 620004503, 620004503, 620004504, 620004504, 620004504, 616140099, 620002885, 620004096, 620004096, 620005638, 621681102, 621681102, 621694602, 621693303, 621694201, 621675901, 616140101, 616140101, 616140104, 616140104, 620003921, 620003921, 620003918, 620003919, 620003919, 620003919, 620003917, 620003917, 620003917, 620003920, 620003922, 620003922, 620003916, 620003916, 620003916, 620003916, 620003923, 620003945, 620004075, 620004075, 620005425, 620005425, 620005425, 620006669, 620006669, 622079301, 621741203, 621741203, 621752801, 621752801, 616140102, 616140102, 616140105, 620003932, 620003932, 620003928, 620003926, 620003926, 620003926, 620003929, 620003929, 620003929, 620003927, 620003927, 620003927, 620003927, 620003930, 620003931, 620003931, 620003933, 620003933, 620003934, 620003935, 620003946, 620004076, 620004076, 620006670, 620006670, 620008013, 620008013, 622079401, 621742103, 621742103, 621742103, 621752901, 621752901, 620003940, 620003941, 620003943, 620003943, 620003943, 620003943, 620003943, 620003942, 620003939, 620003939, 620003939, 620003939, 620003939, 620004077, 620004077, 620004077, 620004476, 620004476, 620004974, 620005426, 620005427, 620006671, 620006671, 620008014, 621753001, 621348401, 621348401, 622295301, 622303301, 622303301, 622269501, 622274301, 622274301, 622274501, 622274501, 622353101, 622411501, 610443026, 610443026, 610443026, 622286701, 622294801, 622294801, 622290801, 622274201, 622274201, 622276801, 622296901, 622303401, 622303401, 622269601, 622275601, 622295401, 622289801, 622289801, 622274401, 622282801, 622281501, 622270801, 622275401, 622275401, 622352901, 622368001, 610451034, 622274601, 622281601, 622270901, 610443024, 610443024, 622290701, 622303201, 622303201, 622269401, 622353001, 620009094, 622085701, 621122501, 621122601, 621122801, 616150003, 616150002, 621123301, 620006083, 620006084, 620007109, 620007109, 621124301, 616150088, 616150088, 621124603, 621124801, 621125101, 616150064, 621125401, 621125701, 616150063, 616150063, 610454083, 610454083, 621126003, 620004770, 622329300, 620005226, 620007513, 621126801, 621126501, 620004918, 616220021, 620004291, 616220017, 616220005, 616220036, 616220036, 616220006, 616220037, 616220037, 616220002, 620008287, 621134201, 621134301, 622341501, 620008358, 646220001, 616220025, 620008333, 620008649, 616220010, 610454007, 610454007, 616220011, 610454008, 610454008, 620003244, 620008620, 620008620, 620004093, 620004093, 620004093, 620004093, 620004093, 620008583, 621127501, 620007375, 620008440, 620007539, 621130301, 621130301, 621131001, 646190020, 646190018, 610462048, 610462049, 622029101, 622289101, 622289201, 622289301, 616210045, 620006826, 616210040 |
| antiviral agent | 620005140, 610444011, 610444011, 620003456, 621444601, 621444601, 621444801, 621444905, 621527604, 621527604, 621720601, 621720601, 622024801, 622024801, 610406386, 610453095, 610453095, 610453095, 610453095, 610453095, 621445001, 621445001, 610444012, 610444012, 610444112, 610444112, 610444112, 610444117, 610444117, 610453001, 610461001, 620009298, 620009298, 621142001, 621353002, 621445205, 621445301, 621445603, 621445701, 616290163, 610433006, 610444013, 610444113, 610444113, 610444113, 610453002, 610453104, 610453104, 610453104, 620003457, 620009299, 620009299, 620009300, 621353802, 621353901, 621445904, 621446001, 621446403, 621446501, 616250001, 610453009, 610453009, 610453009, 610453010, 610453010, 610453010, 610453096, 610453096, 610453096, 621633701, 610463007, 621676401, 610443081, 610443082, 620000425, 620000426, 620004975, 610412192, 610412193, 620004347, 620003516, 622054801, 622054801, 621143601, 621143601, 621143701, 621143701, 610421341, 620004998, 620006943, 621932401, 610462012, 610462012, 622292601, 622281701, 622271501, 622286101, 610443030, 622266101, 622304501, 622304501, 622286801, 622291701, 622295101, 622297701, 622287801, 622276901, 622292701, 622297001, 622301701, 622302701, 622269901, 622276101, 622304301, 622277801, 622298701, 622269301, 622287401, 622287401, 622268601, 622295901, 622293201, 622304401, 622283301, 622281801, 622271401, 622295201, 622273001, 622286501, 622275501, 622275501, 622285501, 610443041, 610443074, 610462002, 620004852, 622083401, 622083401, 610451031, 620000454, 620000455, 620001903, 622336301, 620002413, 620002414, 620002465, 620002488, 620004355, 620005884, 622276701, 622403501, 620006802, 620007815, 620009086, 620009087, 622105001, 622105001, 622149101, 622279401, 622336201, 622363601, 622363501, 622374101, 622418801, 621144201, 621765701, 621384201, 621384201, 621657001, 621662301, 622235801, 622388001, 622408801, 622442101, 622445801, 620003679, 621144401, 620003671, 621384303, 622325900, 620006284, 620006283, 620001341, 620004633, 620004634, 620004634, 620009268, 621144901, 621384402, 621384411, 621384414, 621384424, 640461002, 620003746, 621660102, 620003761, 620003765, 620003765, 622197301, 622197401, 621972202, 621972102, 621972102, 620006495, 620006495, 620006495, 620006496, 620007574, 620008961, 620009008, 620009008, 621995901, 621146701, 621146701, 621146701, 621354901, 621354901, 621146601, 621146601, 621146502, 621146502, 660453001, 620006404, 666250002, 620000360, 620000360, 621447603, 621447501, 660453038, 660453038, 620002305, 620002305, 620002305, 620003042, 660443018, 622012101 |
| antifungal agent | 620008666, 620007031, 620004560, 620004560, 620007032, 620007032, 620007467, 620003487, 620002493, 620002494, 622375301, 640462040, 640462041, 620003489, 620002497, 622136201, 622136301 |
| antiprotozoal agent | 616410003, 616410017, 616410025, 616410025, 616410018, 621166401, 621166501, 620006817, 620007057, 620002484, 622224801, 622225701, 621167101, 622364001, 646410002 |

Appendix 5. Vasopressor codes

| **Types of vasopressors** | **DPC codes** |
| --- | --- |
| vasopressin | 620009273, 642410016 |
| adrenaline | 620517902, 642450005, 621371901, 620518102, 662450001 |
| noradrenaline | 620008384, 642450071 |

Appendix 6. Fluid codes

| **Types of fluids** | **DPC codes** |
| --- | --- |
| normal saline | 620006632, 620006632, 620006632, 620006632, 620006632, 620009265, 620009265, 620009265, 620765904, 643310181, 643310415, 620001325, 620001325, 620001325, 620004323, 620006623, 620006623, 620006623, 620006623, 620006623, 620006625, 620006625, 620766006, 620766024, 620766034, 620766035, 640421007, 640421007, 640421007, 640421007, 640421007, 640421007, 640421007, 640441011, 640441011, 640441011, 640441011, 640441013, 643310182, 643310190, 643310421, 620006627, 620766728, 640441015, 640441015, 643310183, 643310225, 643310434, 643310434, 620006628, 643310184, 640441016, 640441016, 643310185, 643310226, 620006622, 620006622, 620006629, 620006629, 640421008, 640441017, 640441017, 640441017, 643310187, 643310445, 643310445, 643310445, 620006630, 620006630, 640421010, 643310188, 643310452, 620006237, 620767302, 640421009, 640460009, 643310209, 643310209, 643310209, 643310209, 643310286, 643310450, 643310450, 620006238, 640421011, 640460010, 643310210, 643310210, 643310287, 643310454, 643310454, 643310454, 620006626, 620767506, 643310335, 643310356, 643310429, 643310429, 640412105, 640441019, 620004137, 620006236, 620767902, 640407056, 640412107, 643310476, 620000237, 620001328, 621560603, 620000238, 620002471, 620006624, 620006624, 620006624, 620000239, 620006631, 643310473, 643310508, 643310508, 640406034, 640407051, 640407051, 620768401, 640412109, 620002215, 620002215, 620002215, 620008529, 620008858, 622130701, 621672202, 620002216, 620002216, 620008530, 620008859, 620008859, 622132801, 621672103, 620007245, 620004136, 620004136, 620008857, 620009562, 620009562, 620009562, 622130601, 621672303, 620008176, 621956601, 622133001, |
| balanced crystalloid | 643310102, 643310157, 620006274, 620006274, 643310347, 643310328, 620006259, 620006259, 621514701, 621514801, 643310158, 643310348, 643310329, 620006260, 621514901, 643310223, 643310497, 620006258, 643310155, 643310086, 643310086, 643310156, 643310284, 620006253, 620007532, 620006252, 620007531, 620007533, 643310384, 643310336, 640412045, 640412045, 640463101, 620006257, 620007535, 620006256, 643310214, 643310214, 643310213, 643310213, 643310330, 643310391, 620005682, 620005682, 620006255, 620007495, 620005681, 620005681, 620006254, 620007494, 643310394, 640406207, 620008404, 643310170, 620006766, 643310397, 643310493, 643310493, 643310405, 643310486, 643310494, 643310494, 620793902, 620002893, 620005703, 620005703, 620006249, 620008949, 620008949, 620006248, 640412033, 620005702, 620005702, 622121201, 622100901, 622132901, 622127701, 622127701, 622066102, 620795601, 640407166, 620796302, 620006246, 620006247, 620797301, 621311001, 621311101, 620007444, 620007444, 622016601, 622016701 |
| hydroxyethyl starch | 620769401, 620769401, 622250401, 620791601, 620791601 |
| albumin solution | 620008812, 620008812, 620004127, 620008813, 620008813, 620009135, 622042701, 622042701, 621755301, 646340469, 646340469, 646340469, 646340469, 620003721, 621155201, 621155201, 621155501, 620008814, 620008814, 646340472, 646340472, 646340472, 646340472, 620002196, 620008815, 620008815, 620009136, 621450201, 621450201, 621645901, 646340474, 646340474, 646340474, 646340474, 620003722, 620009137, 621157301, 621157301, 621157401 |
| red blood cell transfusion | 646340286, 646340285, 622191101, 622191201, 646340048, 646340226, 621772801, 621772901, 646340242, 646340243, 622190901, 622191001, 621772001, 621772101, 622191701, 622191801, 622191501, 622191601 |

Appendix 7. ICU-10 codes directly indicate “sepsis”

| **ICD-10 codes** |
| --- |
| A02.1, A20.7, A22.7, A26.7, A32.7, A40, A40.0-A40.3, A40.8, A40.9, A41, A41.0-A41.5, A41.8, A41.9, A42.7, B37.7, O85, O88.3, P36, P36.0-P36.5, P36.8, P36.9 |
